# Genotyping and phylogenetic placement of *Bacillus anthracis* isolates from Finland, a country with rare anthrax cases

**DOI:** 10.1186/s12866-018-1250-4

**Published:** 2018-09-03

**Authors:** Taru Lienemann, Wolfgang Beyer, Kirsti Pelkola, Heidi Rossow, Alexandra Rehn, Markus Antwerpen, Gregor Grass

**Affiliations:** 1 Finnish Food Safety Authority (Evira), Veterinary Bacteriology and Pathology Research Unit, Helsinki, Finland; 2 University of Hohenheim, Stuttgart, Germany; 3 Finnish Food Safety Authority (Evira), Risk Assessment Research Unit, Helsinki, Finland; 4 Bundeswehr Institute of Microbiology, Munich, Germany

**Keywords:** *Bacillus anthracis*, Finland, Whole genome sequencing (WGS), Comparative genomics, Single nucleotide polymorphism (SNP), Multiple locus variable number of tandem repeat analysis (VNTR, MLVA)

## Abstract

**Background:**

Anthrax, the zoonotic disease caused by the gram-positive bacterium *Bacillus anthracis*, is nowadays rare in northern parts of Europe including Finland and Scandinavia. Only two minor outbreaks of anthrax in 1988 and in 2004 and one sporadic infection in 2008 have been detected in animals in Finland since the 1970’s. Here, we report on two Finnish *B. anthracis* strains that were isolated from spleen and liver of a diseased calf related to the outbreak in 1988 (strain HKI4363/88) and from a local scrotum and testicle infection of a bull in 2008 (strain BA2968). These infections occurred in two rural Finnish regions, i.e., Ostrobothnia in western Finland and Päijänne Tavastia in southern Finland, respectively.

**Results:**

The isolates were genetically characterized by PCR-based methods such as multilocus variable number of tandem repeat analysis (MLVA) and whole genome-sequence analysis (WGS). Phylogenetic comparison of the two strains HKI4363/88 and BA2968 by chromosomal single nucleotide polymorphism (SNP) analysis grouped these organisms within their relatives of the minor canonical A-branch canSNP-group A.Br.003/004 (A.Br.V770) or canonical B-branch B.Br.001/002, respectively. Strain HKI4363/88 clustered relatively closely with other members of the A.Br.003/004 lineage from Europe, South Africa, and South America. In contrast, strain BA2968 clearly constituted a new sublineage within B.Br.001/002 with its closest relative being HYO01 from South Korea.

**Conclusions:**

Our results suggest that Finland harbors both unique (autochthonous) and more widely distributed, common clades of *B. anthracis*. We suspect that members of the common clades such as strains HKI4363/88 have been introduced only recently by anthropogenic activities involving importation of contaminated animal products. On the other hand, autochthonous strains such as isolate BA2968 probably have an older history of their introduction into Finland as evidenced by a high number of single nucleotide variant sites in their genomes.

**Electronic supplementary material:**

The online version of this article (10.1186/s12866-018-1250-4) contains supplementary material, which is available to authorized users.

## Background

The zoonosis anthrax is often referred to as a neglected tropical disease [1]. This is because most animal and human cases occur in (sub)tropical countries of Sub-Sahara Africa and parts of Asia [2]. However, in the past the bacterium causing anthrax, *Bacillus anthracis*, had a much wider distribution. For instance, high latitude countries such as Sweden, Finland, Canada or the area of the Russian Federation (i.e., the former Soviet Union) had also been affected by the disease, regularly. In Finland, altogether 283 anthrax cases in 150 different localities have been detected in several animal species since 1940’s (Zoonoses in Finland in 2000–2010. online available: https://www.evira.fi/globalassets/elaimet/zoonoosikeskus/zoonoosit/zoonosesinfinland_final_nettiversio.pdf. Accessed July 11, 2018). The majority of anthrax outbreaks involved bovines, but also other animal species such as horses, swine, sheep, dogs and fur animals like minks and foxes have been affected. Due to specific control measurements, improved feed hygiene and awareness of the epidemiology of anthrax, the disease is nowadays rare in Finland. However, *B. anthracis* still causes large outbreaks elsewhere at high latitudes. For instance, after the last outbreak in 1941 in a northern, boreal part of what is now the Yamal-Nenets autonomous district located within the Russian Federation, 2.657 infected animals were reported from six different outbreaks in the summer of 2016. From these animals and their products several human fatalities had resulted in the aftermath [2]. Besides, several outbreaks occurred among farm animals in southeastern Sweden in the same summer [2]. In Canada, anthrax is still enzootic in central parts of the country and free-roaming Wood Bisons are frequently affected during summer months [3].

While there is an increasing number of available genome sequences of *B. anthracis* from broad geographic origins, there are still only a few sequenced isolates from higher latitudes. From Norway, only two multilocus sequence typed bovine isolates originating from two outbreaks in 1987 and 1993, respectively [4], and a few genomes from bovine isolates partly lacking further meta-data have been reported [5, 6]. Conversely, a prominent Norwegian *B. anthracis* genome is the one originating from imported contaminated heroin; the isolate causing the very first fatality of injectional anthrax in 2000 [7]. From Sweden stems one genome and very closely related isolates from a primary outbreak at a nature reserve in 2011. This outbreak was likely caused by the disturbance of a historical animal burial site and resulted in a secondary outbreak in 2013 [8]. Other strains originate from older collections as in the case of Denmark [9] or Finland (this study) because nowadays anthrax is a very rare disease in these countries, too.

In this study, we genotyped and whole genome-sequenced the only two available *B. anthracis* isolates from Finland. One strain (HKI4363/88) originated from an outbreak in Ostrobothnia, western Finland, in August 1988 [10]. Isolate BA2968, was isolated in Helsinki in September 2008 from an aspirate of an oedematous scrotum of a young bull (born in 2007) which was diagnosed with a testicle and scrotum inflammation.

The new genomic data from the Finnish isolates was used to compare these *B. anthracis* isolates with their close relatives and to assign the Finnish strains’ placements in a phylogenetic context.

## Methods

### Growth of *B. anthracis* and extraction of DNA

Vegetative cells of *B. anthracis* from our strain collections were cultured on blood agar, subsequently inactivated and DNA was isolated as described previously [11] using DNeasy Blood and Tissue kit (Qiagen, Germany) as described for Gram-positive bacteria with the following minor modifications. After cell wall lysis with lysozyme (20 mg/ml), 4 μl of RNase A (100 mg/ml) was added and the suspension was incubated for 2 min at room temperature. DNA was eluted twice (50 μl and 50 μl) using sterile nuclease-free water. Handling of live *B*. *anthracis* occurred in a biosafety level 3 laboratory and the isolated DNA was sterile-filtered (0.22 μm filter pore, Merck Millipore, Germany) prior to being taken from biosafety level 3 laboratory. DNA concentrations were quantified using the Qubit dsDNA HS Assay Kit (Thermo Fisher Scientific, USA) according to the supplier’s protocol. DNA preparations were stored at − 20 °C until further use.

### Diagnostic real-time PCR for chromosomal and plasmid markers of *B. anthracis*

Three specific genetic markers including chromosomal marker *dhp61* (BA_5345) as well as plasmid markers *pagA* (pXO1) and *capC* (pXO2) were used to identify *B. anthracis* by real-time PCR as described in [12, 13]. Real-time PCR assays were conducted using a LightCycler 480 II (Roche, Germany) and data analysis was performed with the associated instrument software.

### Analysis of canonical single nucleotide polymorphisms (canSNPs)

Isolates were grouped into the canonical SNP (canSNP) typing scheme that allows attribution into genetic groups within the accepted global population structure of *B. anthracis* [14]. For this, Mismatch Amplification Mutation Assays (Melt-MAMA) [15] for 12 canSNP groups of *B. anthracis*-isolates were performed (primer sequences in Additional file 1) on a LightCycler 480 II instrument (Roche, Germany) as described in [13] and data analysis was performed with the associated instrument software.

### Multi locus variable number of tandem repeats analysis using 31 markers (MLVA-31)

The MLVA was performed essentially as described in [16]. Briefly, amplification of the fragments of 31 marker-loci was performed in 7 multiplex-PCRs (the origins of the best matches to the Finnish *B. anthracis* isolates are listed in Additional file 2). The fragment-mixtures were analyzed on a Genetic Analyzer (ABI 3130, Applied Biosystems, Germany) using either MegaBACE TMET (GE Healthcare, Germany), Genescan 1200 LIZ (Applied Biosystems, USA) or MapMarkerH 1000 (BioVentures, USA) as size standards. The data were analyzed with GeneMapper TM software (Applied Biosystems, USA). The raw data of fragment lengths were normalized by codes, reflecting the actual copy numbers of the repeat sequences where possible.

### Whole genome sequencing and assembly

The Nextera® XT DNA Library Preparation kit (Illumina, USA) was used for library preparation with DNA inputs of 1 to 3 ng per library. Libraries were sequenced on a MiSeq instrument (Illumina, USA) using MiSeq Reagent Kit v3 (600-bp) chemistry (Illumina, USA). High-quality paired-end reads (Q ≥ 30) were assembled de novo into draft genomes using an in-house script based on the SPAdes assembler (version 3.11.1) [17]. The automated genome refining tool Pilon (version 1.22) [18] was used to further improve these draft genomes by correcting SNPs or closing small gaps. All processed scaffolds were manually checked for contaminant reads and uploaded to the NCBI Bioproject database (The data-set supporting the conclusions of this article is available in the Bioproject PRJNA309927, accession numbers: Additional file 3). Annotation was automatically performed by the NCBI Prokaryotic Genome Annotation Pipeline [19].

### Analysis of whole genome sequencing data – SNP calling

The Parsnp tool from Harvest Suite was used for rapid core chromosome multiple-alignment [20]. Input data for this were representative *B. anthracis* genomes from public databases (Additional file 3) and newly sequenced strains that were aligned against the *‘Ames ancestor’* reference chromosome (NC_007530) using Parsnp (parameters -c -e -u -C 1000). Called SNPs were extracted into a VCF file using HarvestTools (version 1.0) from the same software suite. To enhance overall data quality, SNP positions with a distance of less than 10 bp as well as positions harboring an undefined nucleotide (“N”) were removed. The “R” analysis package phangorn (version 2.4.0) was used to determine homoplasious SNPs (parameter “CI(tree, data, cost = NULL, sitewise = TRUE)”) and to remove them [21]. This edited file served again as an input file in the HarvestTools to compile a FASTA file comprising a multiple-sequence alignment of the concatenated SNPs. Next, the evolutionary history was inferred from this data by using the Maximum Likelihood method according to the Tamura-Nei model [22]. A phylogenetic maximum likelihood tree was computed in Mega7 [23] and a minimum spanning tree was computed in BioNumerics 6.6 (Applied Maths, Belgium) from the VCF SNP-file (in binary format) as input and manually edited for style.

## Results

### Genotyping of two *B. anthracis* isolates from Finland

Isolates HKI4363/88 and BA2968 were classified within the three major branches A, B and C and assigned to canonical SNP-groups of *B. anthracis* [14]. HKI4363/88 belonged to the A-branch (A.Br.) A.Br.003/004 defined by an ancestral SNP state for SNP A.B.03 and a derived state for A.Br.004. According to a recently amended nomenclature, this major branch is now called A.Br.V770 defined by the SNP-states of A.Br.003 and A.Br.054 [24]. Isolate BA2968 grouped within the B-branch, B.Br.001/002, typically comprising European and African strains [14].

**Fig. 1:**
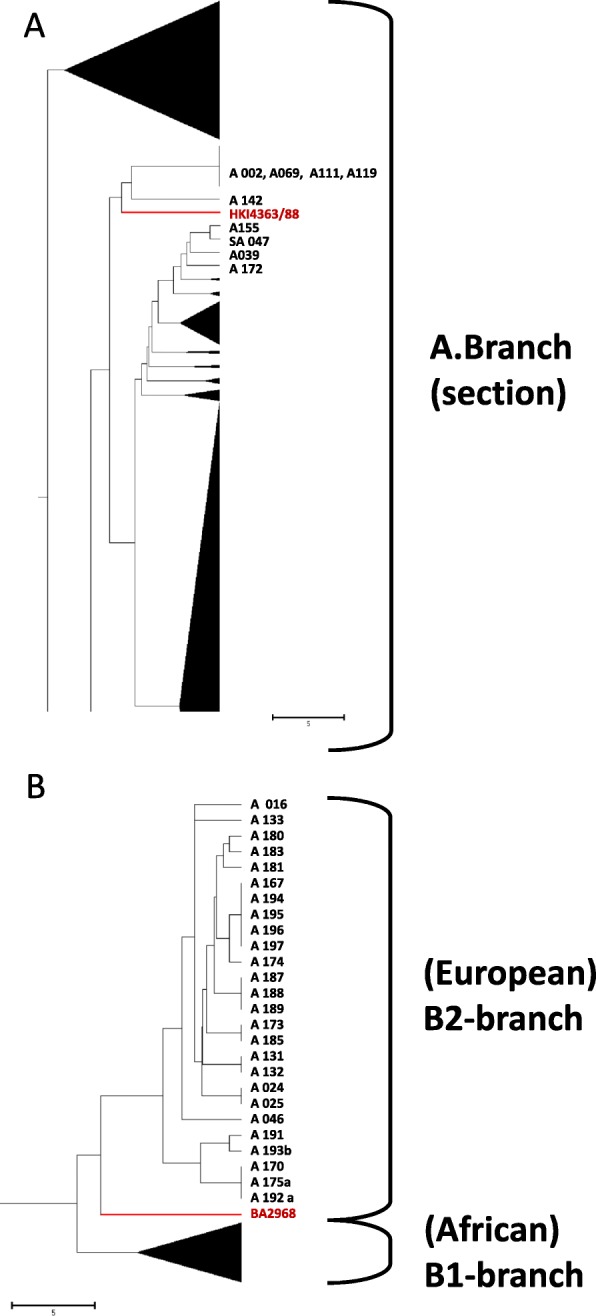
Position of strains HKI4363/88 and BA2968 in UPGMA cluster analysis based on 31 *B. anthracis* MLVA markers. Positions of strains HKI4363/88 (**a**) and BA2968 (**b**) (indicated in red) among their closest MLVA-relatives within a data-set of 976 *B. anthracis* isolates are shown

In order to identify possible close relatives of these Finnish strains, we performed MLVA-31 typing and compared the strains’ profiles with that of our in-house MLVA-database. Additional file 4 summarizes the results of MLVA-31 for strains HKI4363/88, BA2968 and their closest matches. Next, we used this data to visualize the MLVA-similarities between Finnish strains and relatives from other geographical origins (Fig. 1). Finnish isolate HKI4363/88 clustered closely (6 markers difference) with a strain isolated around Cape Town (Rondebosch) in 1999. Near relatives of this strain HKI4363/88 (five markers difference) had also been found in a soil sample of a derelict tannery (Neumünster, Germany) with a > 100 year history, namely isolate A155. Isolate A172 originated from an outbreak in bovines (four markers difference) in France in the year 2001. Interestingly, there is also a more distant relation (seven markers difference) to the *B. anthracis* type strain V770 NP/1 (ATCC 14185) used for human vaccine production in the USA for a long time. Another relative of this group (A142) was found at the same tannery site as strain A155. From the MLVA-31 results presented in Fig. 1, we selected seven strains from our strain collections most similar in their VNTR-profile to Finnish isolates HKI4363/88 or BA2968, respectively, for whole genome sequencing (Additional file 3).

### Chromosomal SNP analysis suggests that Finnish *B. anthracis* strains comprise both an autochthonous lineage and introduction via more recently importation events

Genome sequencing of the two Finnish isolates HKI4363/88 and BA2968 as well as strains A16, A24, A46, A142, A155 and SA047 yielded an average number of 612,000 reads (588,000 – 660,000) per isolate, resulting in an average sequencing depth of > 52-fold. De novo assembly produced between 22 and 41 scaffolds (> 500 bp) per genome. Each isolate covered at least 99.95% of the reference chromosome of *B. anthracis* str*.* ‘Ames ancestor’ (NC_007530).

**Fig. 2:**
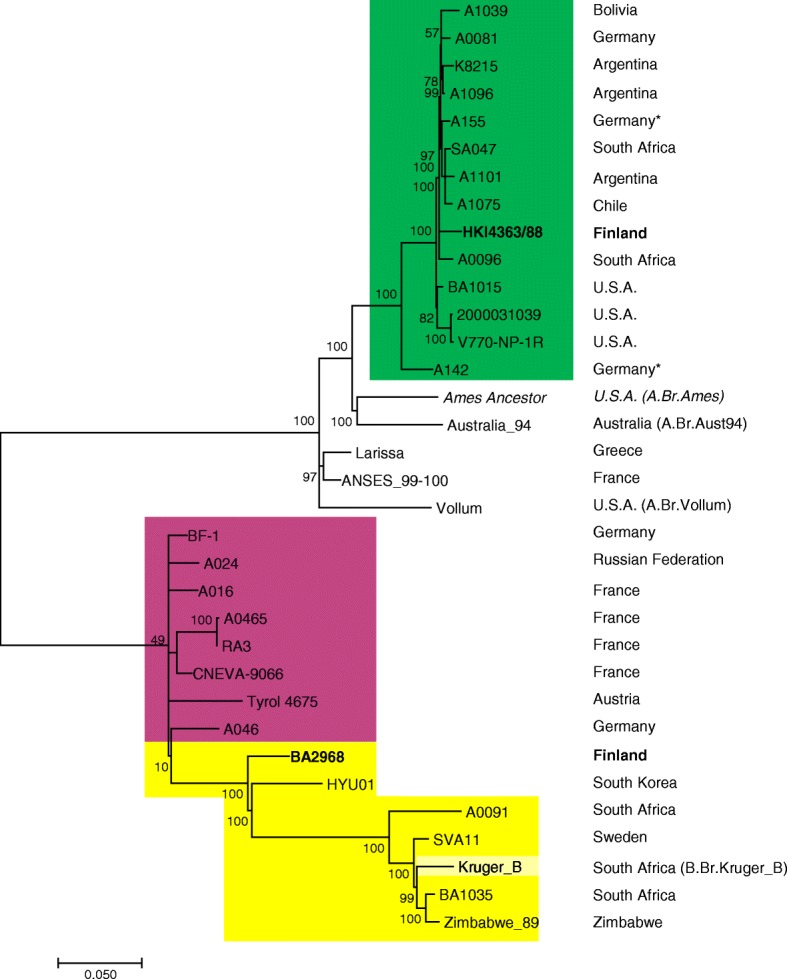
Unrooted phylogenetic tree of representative *B. anthracis* strains derived from chromosomal SNPs. The evolutionary history of *B. anthracis* strains was inferred from 1,548 non-homoplasious chromosomal SNPs by using the Maximum Likelihood method based on the Tamura-Nei model [22]. The tree is drawn to scale, with branch lengths measured in the number of substitutions per site. Colors indicate canSNP groups A.Br.003/004 (A.Br.V770, green), B.Br.CNEVA (purple) or B.Br.001/002 (yellow), respectively. Bootstrap values (based on 1000 replications) are given as percentages at branch nodes. The right column indicates the country of origin and canSNP group information of representatives (in brackets)

From this chromosomal dataset of the in-house sequenced strains and representatives from public databases (Additional file 3) 3211 non-homoplasious SNPs were called of which 1548 were parsimony informative sites (Additional file 5). The concatenated chromosome-wide SNPs were used to infer the phylogenetic relationships of the analyzed strains with focus on the A.Br.003/004 and all three B-lineages (Fig. 2). Chromosomes from strains of neighboring canSNP groups A.Br.Ames, A.Br.001/002 Sterne and A.Br.003/004 V770 were included for reference. Within the phylogeny, strain HKI4363/88 clustered relatively closely with other members of the A.Br.003/004 lineage including strains from Germany, South Africa, Argentina, Bolivia, and Chile and more loosely with isolates from the United States of America. Notably, the other Finnish strain BA2968, positioned within canSNP group B.Br.001/002 as the sole member of a sister clade to the one leading to strains from South Korea, South Africa, Zimbabwe and Sweden as well as including the branch leading to canSNP group CNEVA (Fig. 2).

When focusing on SNP-differences between isolated strains it became obvious that strain BA2968 was unique, clearly constituting a new sublineage within B.Br.001/002. This isolate was separated by 215 SNPs to its closest relative, HYO01, from South Korea and 256 SNPs to canSNP-group B.Br.CNEVA isolate BF-1 from Germany (Additional file 6). Conversely, strain HKI4363/88 was separated by only 52 SNPs distance to strain A1096 from South Africa and less than 100 SNPs to a cluster of isolates from Germany, Bolivia, Argentina, Chile and the United States of America. Of note, the German strains from this group were isolated from the site of a derelict tannery and thus have been likely imported via contaminated animal products (W. Beyer, unpublished).

## Discussion

The two isolates from Finland offer a rare glimpse into the past genomic diversity of *B. anthracis* in European high northern latitudes. During the small outbreak in western Finland in 1988, from which strain HKI4363/88 was isolated, two out of 14 bovines, one heifer and one-year-old calf were infected with *B. anthracis* with typical sudden anthrax symptoms of tremble, fever, paralysis and bleeding. Prior death the calf was treated with penicillin and was bleeding from the anus. Both animals died a couple of hours after the onset of the symptoms. Anthrax was suspected by a municipal veterinarian and *B. anthracis* was isolated from liver and spleen and confirmed by Giemsa staining, animal experiment, and later by PCR at EVIRA (Finnish Food Safety Authority). At the same farm where the two diseased animals were kept in 1988, two earlier cases of anthrax had been documented in 1986. Furthermore, within the immediate surrounding of the farm, anthrax had been documented 30 years earlier [10].

Strain BA2968 was sampled from a bull examined at Saari clinic at the production animal hospital of the University of Helsinki, where bacteria belonging to the genus *Bacillus* were detected in the scrotum aspirate culture. The *Bacillus* isolate was examined further at EVIRA by PCR and identified as *B. anthracis.* The bull was culled immediately after the diagnosis. Blood samples of all the cows with fever from the same farm were examined for *B. anthracis* with negative results. At the same farm where the bull was kept, two cows had died of anthrax 4 years earlier. In addition, diseased bovine animals had been buried in the area several decades ago and also a factory rendering animal byproducts had been run many years ago upstream of a ditch flowing into the farm area. This factory had also processed imported material of animal origin and possibly contaminated the surroundings. Thus, the *B. anthracis* strain causing this “cutaneous” anthrax was probably introduced into the scrotum tissue through a skin wound or abrasion by contaminated soil material after a very rainy summer and autumn. Because of the close vicinity of anthrax outbreaks to the factory processing also imported animal byproducts, it is possible that isolate BA2968 does not represent an autochthonous *B. anthracis* strain but one that has been anthropologically introduced.

In northern latitude countries such as Finland and Sweden, anthrax control programs of the past have resulted in an increasingly rare occurrence of the zoonosis today. In Sweden, a ban on the import of bone meal for animal feed in 1957 led to a steep decline in new outbreaks within a very few years [25]. In the last half century, only outbreaks in 1981, 2008, the most likely linked outbreaks of 2011/13 and a last one in 2016 were documented [8, 25]. Thus, characterization of isolates from such rare events can provide unique opportunities to record remnants of the genetic diversity of the all but extinct pathogen in this country. Possibly, in Sweden and Norway as well, there will be a situation similar to that in Finland with *B. anthracis* genotypes recovered that may be either autochthonous or originate from importation of contaminated animal products such as wool, hides or bone meal.

Taking the phylogeography of *B. anthracis* isolate BA2968 into account, our data indicates a common origin of this and other strains belonging to the so-called (European) B2-Branch [26] isolated in central Europe. The European B-Branch, as originally specified by MLVA, is a wide-spread group of *B. anthracis* strains which are most probably of African origin [27].

## Conclusions

There is some information on the genomic diversity on *B. anthracis* from higher northern latitudes. Nevertheless, it would be worthwhile to mine the genomes of further isolates present in older collections of this pathogen. Such efforts would advance our capabilities in differentiating natural outbreaks in geographic locations where anthrax is very uncommon from deliberate releases of the pathogen. With that kind of information it will then be possible to conduct bioforensically sound outbreak related trace-back analysis and attribution.

## Additional files

Additional file 1: Melt-MAMA canSNP primer sequences. (XLSX 11 kb)

Additional file 2: Origin of near relatives of strain HKI4363/88 and strain BK2968. (XLSX 9 kb)

Additional file 3: Genome sequences accession numbers of Finnish and additional *B. anthracis* strains from publically available databases. (XLSX 11 kb)

Additional file 4: MLVA-31 matrix of strains HKI4363/88 and BA2968 and their closest matches. (XLSX 22 kb)

Additional file 5: Chromosome-wide binary SNP matrix of analyzed genomes. (XLSX 530 kb)

Additional file 6: Position and SNP-distances of Finnish *B. anthracis* strains within their relatives in a Minimum Spanning Tree based on chromosomal SNPs. A Minimum Spanning Tree was inferred from 1,548 non-homoplasious chromosomal SNPs using the same dataset as in Fig. 2. Numbers next to branch lines indicate SNPs separating nodes or strains. The coloring is the same as in Fig. 2. (PDF 153 kb)

## Abbreviations

bp: Base pairs
canSNP: Canonical Single Nucleotide Polymorphism
CNEVA: Centre National d’Etudes Veterinaires et Alimentaires
DDBJ/ENA: DNA Data Bank of Japan/ European Nucleotide Archive
DNA: Deoxyribonucleic acid
EVIRA: Finnish Food Safety Authority
FASTA: FAST All
MAMA: Mismatch amplification mutation assay
MLVA: Multiple locus variable number of tandem repeat analysis
NCBI: National Center for Biotechnology Information
PCR: Polymerase chain reaction
RNAse: Ribonuclease
SNP: Single nucleotide polymorphism
USA: United States of America
VCF: Variant call format
VNTR: Variable number of tandem repeat
WGS: Whole genome sequencing
