# Supplementary figures and images for: Genotyping and phylogenetic placement of Bacillus anthracis isolates from Finland, a country with rare anthrax cases
Source: BMC Microbiol. 2018 Sep 3;18:102. doi: 10.1186/s12866-018-1250-4 (PMC6122712; doi:10.1186/s12866-018-1250-4)

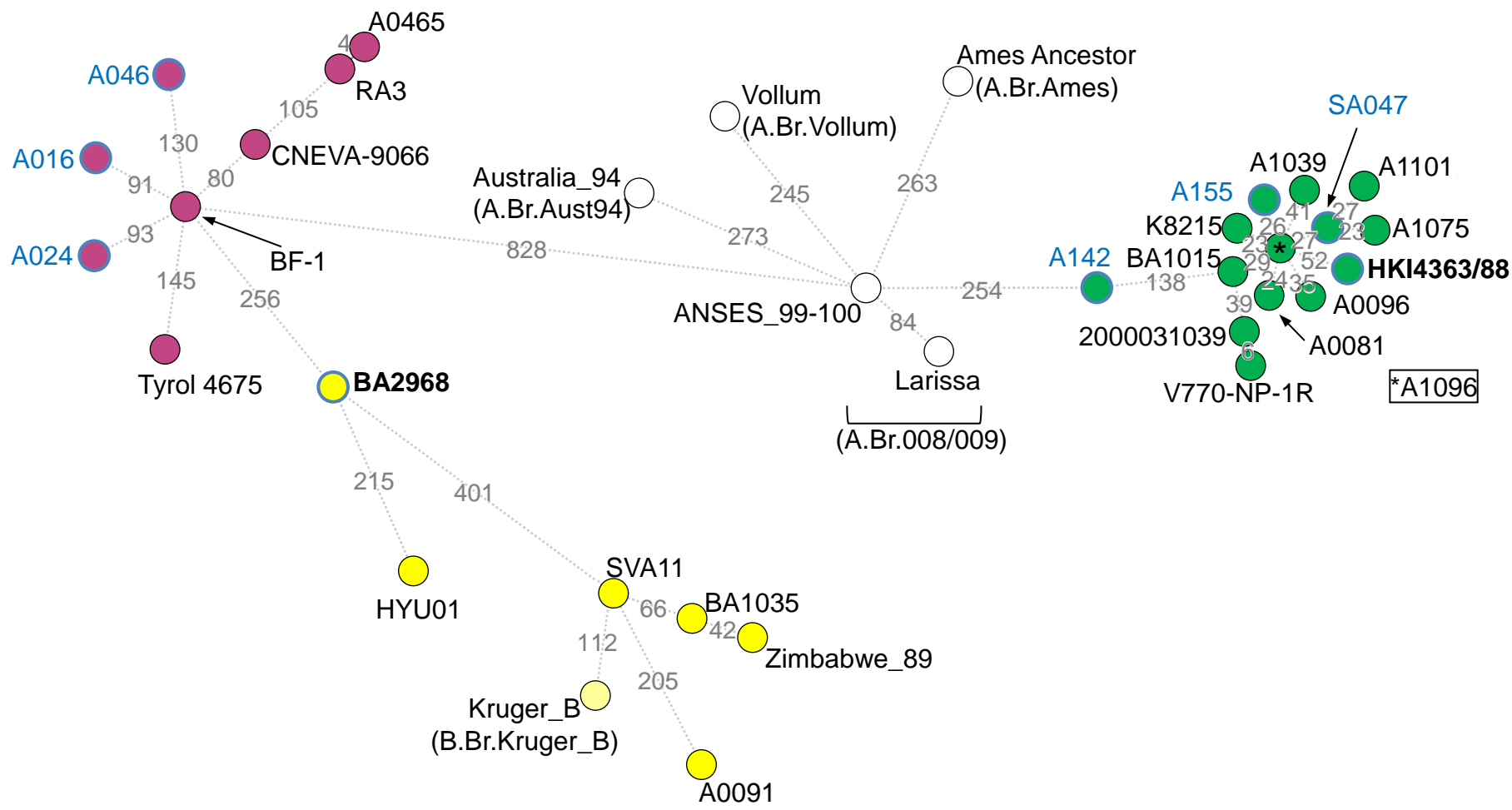

Supplement: Supplementary file 6 — Position and SNP-distances of Finnish B. anthracis strains within their relatives in a Minimum Spanning Tree based on chromosomal SNPs. A Minimum Spanning Tree was inferred from 1,548 non-homoplasious chromosomal SNPs using the same dataset as in Fig. 2. Numbers next to branch lines indicate SNPs separating nodes or strains. The coloring is the same as in Fig. 2. (PDF 153 kb) [file 12866_2018_1250_MOESM6_ESM.pdf]
